# CTAD as a universal anticoagulant

**DOI:** 10.1155/S1463924603000038

**Published:** 2003

**Authors:** M. Yokota, N. Tatsumi, I. Tsuda, T. Nishioka, T. Takubo

**Affiliations:** 1 Department of Clinical and Laboratory Medicine Osaka City University Medical School 1-4-3 Ashinachi Abeno Osaka 545-8585 Japan; 2 Noriyuki Tatsumi International Buddhist University 3-2-1 Gakuenmae Habikino Osaka 583-8501 Japan

## Abstract

The feasibility of CTAD (a mixture of citrate, theophylline, adenosine and dipyridamole) as a new anticoagulant for medical laboratory use was studied prospectively. Whole blood anticoagulated with CTAD exhibited results very similar to those of blood anticoagulated with EDTA on complete blood count and automated white cell differential except for a slight decrease in platelet count and mean platelet volume. Chemistry test data for plasma obtained from CTAD whole blood were close to those obtained for matched sera. Among coagulation tests, prothrombin time, activated partial thromboplastin time and fibrinogen concentrations were close to those obtained with citrate plasma. Based on the results, CTAD was judged to be a good candidate as a new anticoagulant.


Journal of Automated Methods & Management in Chemistry
Vol. 25, No. 1 (January–February 2003) pp. 17–20

Taylor & Francis

Taylor & Francis Group

### CTAD as a universal anticoagulant

**M. Yokota, N. Tatsumi*, I. Tsuda, T. Nishioka and T. Takubo**

*Department of Clinical and Laboratory Medicine, Osaka City University Medical School, 1-4-3 Ashinachi, Abeno, Osaka 545-8585, Japan*

*The feasibility of CTAD (a mixture of citrate, theophylline, adenosine and dipyridamole) as a new anticoagulant for medical laboratory use was studied prospectively. Whole blood anticoagulated with CTAD exhibited results very similar to those of blood anticoagulated with EDTA on complete blood count and automated white cell differential except for a slight decrease in platelet count and mean platelet volume. Chemistry test data for plasma obtained from CTAD whole blood were close to those obtained for matched sera. Among coagulation tests, prothrombin time, activated partial thromboplastin time and fibrinogen concentrations were close to those obtained with citrate plasma. Based on the results, CTAD was judged to be a good candidate as a new anticoagulant.*

### Introduction

The modern medical laboratory consists of fully automated analysers, and in it various types of specimen containers are processed with robotic systems [1]. Patients must supply a lot of blood to fill such containers for stable automated laboratory tests.

A recent trend in laboratory testing is to test multiple items with a minimum amount of sample blood, although the reality is often quite different. To reduce sample blood volume for laboratory testing, various kinds of universal anticoagulants have been proposed, but most are not satisfactory for universal use [2–5]. On the other hand, CTAD, a mixture of sodium citrate, citric acid, theophylline, adenosine and dipyridamole was first developed for use in blood coagulation tests with less effect on coexisting platelets [6, 7].

CTAD blood can be used for coagulation tests. If a haematology test, chemistry and coagulation test were achieved simultaneously from one test tube in an automated laboratory test line, it would be very desirable for patients, since whole blood anticoagulated with heparin has been routinely used for rapid chemistry tests. In this context, the use of CTAD blood was attempted for three kinds of tests: haematology, chemistry and coagulation as a pilot study.

### Experimental

### *Anticoagulants*

CTAD tubes were purchased from Becton-Dickinson Japan (Tokyo, Japan). Vacuum blood collection tubes containing EDTA-2K or sodium citrate were purchased from Terumo Co. (Tokyo, Japan).

### *Specimens*

Venous blood was obtained from adult healthy donors (aged 25–47 years). Multiple specimens with or without an anticoagulant were prepared with the same blood. The specimens were left to stand for 1 h at room temperature and then centrifuged at 1300 $g$ for 20 min to obtain plasma or serum.

### *Haematology tests*

Whole blood specimens ($n = 46$ adult healthy donors aged 25–45 years) were obtained under informed consent. After blood withdrawal, all specimens were kept at room temperature, and all experiments were completed within 6 h of collection. Haematology analysers, the SE-9000 (Sysmex Corp., Kobe, Japan) and a CELL-DYN 4000 haematology system (Abbott Diagnostics, Chicago, IL, USA), were used for complete blood count (CBC) to obtain red blood cell count (RBC), white blood cell count (WBC), whole blood haemaglobin (Hb), haematocrit (Hct), mean cell volume (MCV), red cell size distribution (RDW), platelet count (Plt) and automated white cell differential.

Push-wedge slides were prepared manually and stained with May–Giemsa as controls for evaluation of automated white cell differential.

### *Chemistry tests*

Plasma from CTAD blood ($n = 6$, adult healthy donors aged 30–47 years) were analysed with a fully automated Hitachi 7170 multiple analyser (Hitachi Co., Tokyo, Japan). As controls, serum specimens were prepared. The biochemical and electrolyte items to be measured are shown in table 1.

### *Coagulation tests*

Venous specimens were obtained from 21 adult healthy donors (aged 25–45 years) in two kinds of blood collection tubes containing sodium citrate or CTAD. Separated plasma was tested within 3 h after blood collection. Prothrombin time (PT), activated partial thromboplastin time (APTT), thrombo test (TH-T) and fibrinogen concentration were measured with specific reagents and a CA-6000 coagulation analyser (Sysmex).

### *Statistical analysis*

Statistical analysis was performed using a simple regression analysis with Microsoft Excel 98.

* Author to whom correspondence should be addressed. Noriyuki Tatsumi, International Buddhist University, 3-2-1 Gakuenmae, Habikino, Osaka 583-8501, Japan. e-mail: ntatsumi@mail.shitennoji.ac.jp

*Journal of Automated Methods & Management in Chemistry* ISSN 1463–9246 print/ISSN 1464–5068 online © 2003 Taylor & Francis Ltd
http://www.tandf.co.uk/journals
DOI: 10.1080/1463924031000066212

*Table 1. Methods used for analysis of biochemical and electrolyte parameters.*

| Items | Method |
|---|---|
| Total protein (TP) | Biuret |
| Albumin (ALB) | BCG (bromcresol green) |
| Total bilirubin (T-bil) | alkalineazo bilirubin |
| Blood urea nitrogen (BUN) | urease UV |
| Creatinine (CRE) | alkaline picrate |
| Uric acid (UA) | uricase peroxidase |
| Lactose dehydrogenase (LDH) | Wroblewski–Ladue |
| Aspartate aminotransferase (AST) | UV |
| Alanine aminotransferase (ALT) | UV |
| Glucose (Glu) | glucose oxidase |
| C-reactive protein (CRP) | turbidimetric immunoassay |
| Sodium (Na) | electrode method by Hitachi 710 |
| Chlorine (Cl) | electrode method by Hitachi 710 |
| Potassium (K) | electrode method by Hitachi 710 |

### Results

*Automated haematology tests*

CBC and automated white cell differential were compared between CTAD blood and EDTA-2K blood. In CTAD blood, the proportion of blood to the CTAD solution was 9:1. Therefore, the values of items influenced by dilution were corrected and compared with those values from EDTA blood. Data analyses (means, distribution width and simple regression analysis, $r$ for counts and $p$ are shown in tables 2a and 2b. The means and ranges of all parameters determined from the CTAD samples compared well with those from the EDTA

*Table 2a. Correlation between complete blood count profile parameters for EDTA and CTAD blood measured with SE-9000.*

| | EDTA: $y$* | CTAD: $x$* | $y = ax + b$ | $r$ | $p$ |
|---|---|---|---|---|---|
| RBC ($\times 10^6\ \mu l^{-1}$) | 4.36 ± 0.51 | 4.42 ± 0.52 | $y = 0.981x + 0.020$ | 0.994 | <0.0001 |
| Hb ($g\,dl^{-1}$) | 13.3 ± 1.51 | 13.4 ± 1.60 | $y = 0.945x + 0.615$ | 0.996 | <0.0001 |
| Hct (%) | 40.0 ± 4.26 | 40.8 ± 4.38 | $y = 0.967x + 0.496$ | 0.994 | <0.0001 |
| MCV ($\mu m^3$) | 92.0 ± 6.22 | 92.6 ± 6.22 | $y = 0.997x - 0.288$ | 0.997 | <0.0001 |
| RDW (%) | 12.0 ± 0.95 | 12.0 ± 0.94 | $y = 1.010x - 0.087$ | 0.994 | <0.0001 |
| Platelet ($\times 10^3\ \mu l^{-1}$) | 22.9 ± 7.66 | 21.0 ± 7.17 | $y = 1.044x + 1.013$ | 0.977 | <0.0001 |
| WBC ($\times 10^3\ \mu l^{-1}$) | 6.28 ± 1.92 | 6.24 ± 1.91 | $y = 0.999x + 0.049$ | 0.993 | <0.0001 |
| Neutrophil ($\times 10^3\ \mu l^{-1}$) | 4.00 ± 1.75 | 3.99 ± 1.84 | $y = 0.935x + 0.267$ | 0.981 | <0.0001 |
| Lymphocyte ($\times 10^3\ \mu l^{-1}$) | 1.69 ± 0.71 | 1.72 ± 0.75 | $y = 0.902x + 0.144$ | 0.957 | <0.0001 |
| Monocyte ($\times 10^3\ \mu l^{-1}$) | 0.34 ± 0.11 | 0.27 ± 0.13 | $y = 0.516x + 0.198$ | 0.618 | <0.0001 |
| Eosinophil ($\times 10^3\ \mu l^{-1}$) | 0.21 ± 0.20 | 0.23 ± 0.21 | $y = 0.895x + 0.011$ | 0.988 | <0.0001 |
| Basophil ($\times 10^3\ \mu l^{-1}$) | 0.03 ± 0.02 | 0.03 ± 0.02 | $y = 0.725x + 0.012$ | 0.606 | <0.0001 |

$n = 46$.
* Each value is the mean ± SD.

*Table 2b. Correlation between complete blood count profile parameters for EDTA and CTAD blood measured with CD-4000.*

| | EDTA: $y$* | CTAD: $x$* | $y = ax + b$ | $r$ | $p$ |
|---|---|---|---|---|---|
| RBC ($\times 10^6\ \mu l^{-1}$) | 4.27 ± 0.49 | 4.34 ± 0.51 | $y = 0.958x + 0.116$ | 0.989 | <0.0001 |
| Hb ($g\,dl^{-1}$) | 12.9 ± 1.43 | 13.1 ± 1.47 | $y = 0.961x + 0.288$ | 0.991 | <0.0001 |
| Hct (%) | 40.2 ± 4.16 | 40.9 ± 4.30 | $y = 0.954x + 1.159$ | 0.985 | <0.0001 |
| MCV ($\mu m^3$) | 94.5 ± 6.33 | 94.8 ± 6.27 | $y = 1.006x - 0.828$ | 0.997 | <0.0001 |
| RDW (%) | 13.5 ± 0.97 | 13.6 ± 0.99 | $y = 0.978x + 0.161$ | 0.991 | <0.0001 |
| Platelet ($\times 10^3\ \mu l$) | 23.1 ± 7.89 | 20.5 ± 6.90 | $y = 1.121x + 1.950$ | 0.981 | <0.0001 |
| WBC ($\times 10^3\ \mu l$) | 6.62 ± 1.99 | 6.62 ± 2.05 | $y = 0.964x + 0.243$ | 0.994 | <0.0001 |
| Neutrophil ($\times 10^3\ \mu l$) | 3.94 ± 1.68 | 3.95 ± 1.74 | $y = 0.960x + 0.142$ | 0.997 | <0.0001 |
| Lymphocyte ($\times 10^3\ \mu l$) | 2.05 ± 0.78 | 2.00 ± 0.76 | $y = 1.017x + 0.019$ | 0.988 | <0.0001 |
| Monocyte ($\times 10^3\ \mu l$) | 0.41 ± 0.16 | 0.44 ± 0.17 | $y = 0.740x + 0.082$ | 0.822 | <0.0001 |
| Eosinophil ($\times 10^3\ \mu l$) | 0.21 ± 0.20 | 0.20 ± 0.19 | $y = 1.047x - 0.004$ | 0.984 | <0.0001 |
| Basophil ($\times 10^3\ \mu l$) | 0.01 ± 0.01 | 0.02 ± 0.04 | $y = 0.090x + 0.010$ | 0.246 | 0.099 |

$n = 46$.
* Each value is the mean ± SD.

*Table 3. Correlation between biochemical and electrolyte items for serum and CTAD plasma.*

| | Serum: $y$* | CTAD plasma: $x$* | $y = ax + b$ | $r$ |
|---|---|---|---|---|
| TP ($g\,dl^{-1}$) | $7.0 \pm 0.98$ | $6.9 \pm 0.92$ | $y = 1.045x - 0.263$ | 0.991 |
| ALB ($g\,dl^{-1}$) | $4.1 \pm 0.21$ | $4.1 \pm 0.15$ | $y = 0.876x + 0.482$ | 0.983 |
| T-Bi ($mg\,dl^{-1}$) | $0.6 \pm 0.29$ | $0.7 \pm 0.14$ | $y = 0.813x + 0.065$ | 0.987 |
| BUN ($mg\,dl^{-1}$) | $15.8 \pm 4.46$ | $15.4 \pm 4.66$ | $y = 0.948x + 1.150$ | 0.995 |
| CRE ($mg\,dl^{-1}$) | $0.75 \pm 0.34$ | $0.73 \pm 0.33$ | $y = 1.004x + 0.016$ | 0.999 |
| UA ($mg\,dl^{-1}$) | $4.8 \pm 1.6$ | $4.9 \pm 1.7$ | $y = 0.922x + 0.353$ | 0.995 |
| LDH ($IU\,l^{-1}$) | $422 \pm 135$ | $379 \pm 127$ | $y = 1.021x + 34.53$ | 0.960 |
| AST ($IU\,l^{-1}$) | $34 \pm 16.0$ | $31 \pm 11.3$ | $y = 0.764x + 6.896$ | 0.965 |
| ALT ($IU\,l^{-1}$) | $34 \pm 16.4$ | $34 \pm 16.0$ | $y = 1.017x + 0.929$ | 0.990 |
| CK ($IU\,l^{-1}$) | $135 \pm 115$ | $126 \pm 108$ | $y = 1.064x + 1.233$ | 0.999 |
| Glu ($g\,dl^{-1}$) | $104 \pm 10.1$ | $96 \pm 9.2$ | $y = 1.063x + 1.955$ | 0.962 |
| CRP ($mg\,ml^{-1}$) | $0.3 \pm 0.51$ | $0.3 \pm 0.47$ | $y = 1.078x - 0.004$ | 0.996 |
| Na ($mEq\,l^{-1}$) | $143 \pm 2.26$ | $177 \pm 2.48$ | † | 0.053 |
| Cl ($mEq\,l^{-1}$) | $107 \pm 2.8$ | $96 \pm 4.2$ | $y = 0.336x + 74.87$ | 0.515 |
| K ($mEq\,l^{-1}$) | $4.0 \pm 0.37$ | $3.9 \pm 0.35$ | $y = 1.038x + 0.020$ | 0.980 |

* Each value is the mean ± SD.
† With no correlation.

samples, except for absolute basophil counts and monocyte counts. A good correlation was demonstrated for platelet count, although the value was lower for CTAD blood than for EDTA blood. CTAD blood showed no morphological effects on blood cells and no active platelet aggregation on Giemsa-stained blood films.

### *Automated chemistry tests*

The blood needed dilution correction because the CTAD solution was liquid and diluted blood. As shown in table 3, 13 of 15 biochemical and electrolyte items had coefficients of correlation between the serum and CTAD groups >0.95, while the coefficient of correlation for Na and Cl were low, at 0.2 and 0.5, respectively, because CTAD contains large amounts of sodium.

### *Automated coagulation tests*

Three of five coagulation test items, PT, APTT and fibrinogen concentration, exhibited very high correlation coefficients >0.9 between CTAD plasma and citrate plasma, although HEP-T and TH-T how low correlation coefficients of 0.79 and 0.67, respectively (table 4).

### Discussion

In modern laboratory testing systems, various kinds of specimens are required, such as serum and whole blood anticoagulated with EDTA and sodium citrate, which require much blood to obtain laboratory data, although many researchers and patients also wish to decrease the volume of sample blood for laboratory testing. Recently, many reports have described universal anticoagulants for the reduction of blood volume for laboratory tests.

CTAD was first developed for coagulation tests to eliminate coexisting platelet effects in platelet-poor plasma. CTAD is now also used for the platelet factor 4 and $\beta$-thromboglobulin assays. However, no consideration has been made of its uses as a universal anticoagulant. Reinhart *et al.* [8] first tried to use CTAD blood for haematological testing in a relatively small number of cases, and they reported that no difference in haematological data produced by electronic particle counters was observed between EDTA and CTAD blood. Tsuda *et al.* [9] also tried to use CTAD blood for complete blood count and automated white cell differential to prove its efficacy for haematological analysis. In this study, large numbers of samples were investigated with two automated analyser systems based on different methods. The data for items related to RBC were similar to those in

*Table 4. Correlation between coagulation test items for citrate versus CTAD plasma.*

| | Citrate plasma: $y$* | CTAD plasma: $x$* | $y = ax + b$ | $r$ |
|---|---|---|---|---|
| PT (s) | $11.4 \pm 1.98$ | $10.9 \pm 1.75$ | $y = 1.058x - 0.117$ | 0.937 |
| APTT (s) | $38.6 \pm 5.35$ | $39.0 \pm 5.83$ | $y = 0.889x + 3.967$ | 0.969 |
| TH-T (s) | $89 \pm 46.6$ | $212 \pm 139$ | $y = 0.202x + 37.74$ | 0.670 |
| HEP-T (%) | $88 \pm 26.0$ | $121 \pm 45.5$ | $y = 0.453x + 33.16$ | 0.792 |
| Fibrinogen ($mg\,dl^{-1}$) | $298 \pm 134$ | $298 \pm 98.2$ | $y = 0.920x - 10.68$ | 0.923 |

$n = 20$.
* Each value is the mean ± SD.

EDTA blood and CTAD blood. In RBC morphology, CTAD blood exhibited a shift in shape toward echinocytes [8], but a high degree of correlation was observed in our data for items associated with RBC, including RDW between EDTA and CTAD. The data for items related to WBC are similar for EDTA-2K blood and CTAD blood, except for basophil count. It is thought that the relative scarcity of basophils in normal blood samples is primarily responsible for this. Platelet counts were somewhat lower than those in the EDTA-2K, but this item has a good correlation for CTAD samples and EDTA-2K samples. CTAD blood is thus useful for analysis by automated haematology analyser systems.

As described above, such CTAD blood is useful for automated haematology tests. It would be desirable if such blood could also be used for chemistry tests. We therefore tried to use CTAD blood for a chemistry test and compared it with the test data obtained from serum specimens. The results were in good accordance with chemistry tests. One of the demerits of CTAD blood is that it cannot provide accurate data for sodium and chlorine because the sodium ion is contained in the CTAD solution itself.

In coagulation test items, PT, APTT and fibrinogen concentration exhibited very high correlation coefficients between CTAD plasma and citrate plasma. On the other hand, HEP-T and TH-T exhibited relatively low correlation, although these two items are not in common usage. The results indicated that CTAD blood is useful not only for coagulation and haematology tests, but also for chemistry tests.

In conclusion, we found that CTAD is an excellent candidate for a new anticoagulant usable for simultaneous evaluation of haematological, biochemical and coagulation items in the routine laboratory.

### References

1. Boyd, J. C., Felder, R. A. and Savory, J., *Clinical Chemistry*, **42** (1996), 1901.
2. Perrotta, G., Roberts, L., Glazier, J. and Schumacher, H. R., *Lab. Haematology*, **4** (1998), 156.
3. Kinoshita, Y., Ohta, K., Yamane, T., Hino, M., Takubo, T., Samori, T. and Tatsumi, N., *J. Clin. Lab. Anal.*, **14** (2000), 180.
4. Kumura, T., Hino, M., Yamane, T. and Tatsumi, N., *J. Automated Meth. Management Chem.*, **22** (2000), 109.
5. Kawamoto, T., Hino, M., Takubo, T. and Tatsumi, N., *Osaka City Med. J.*, **46** (2000), 37.
6. Contant, G., Gouault-Heilmann, M. and Martinoli, J. L., *Thromb. Res.*, **31** (1983), 365.
7. van den Besselaar, A. M. H. P., Meeuwisse-Braun, J., Jansen-Grüter, R. and Bertina, R. M., *Thromb. Haemostas.*, **57** (1987), 226.
8. Reinhart, W. H., Haeberli, A., Stark, J. and Straub, P. W., *J. Lab. Clin. Med.*, **115** (1990), 98.
9. Tsuda, I., Katagami, T. and Tatsumi, N., *Med. Biol.*, **140** (2000), 93.

